# Parameter Optimisation of Johnson–Cook Constitutive Models for Single Abrasive Grain Micro-Cutting Simulation: A Novel Methodology Based on Lateral Material Displacement Analysis

**DOI:** 10.3390/ma18245559

**Published:** 2025-12-11

**Authors:** Łukasz Rypina, Dariusz Lipiński, Robert Tomkowski

**Affiliations:** 1 Department of Mechanical Engineering, Faculty of Mechanical and Energy Engineering, Koszalin University of Technology, Racławicka 15, 75-620 Koszalin, Poland; dariusz.lipinski@tu.koszalin.pl; 2 Department of Production Engineering, KTH Royal Institute of Technology, Brinellvägen 68, 114 28 Stockholm, Sweden; rtom@kth.se

**Keywords:** Johnson–Cook constitutive model, single abrasive grain, finite element modelling, strain rate sensitivity, micro-grinding

## Abstract

**Highlights:**

**What are the main findings?**

- The Johnson–Cook models were analysed under grinding conditions;
- Criteria were developed to assess non-conformity cross-section scratches.

**What are the implications of the main findings?**

- A method for optimising the Johnson–Cook constitutive equations was proposed;
- The applied methodology enables high computational accuracy.

**Abstract:**

The accurate modelling of material removal mechanisms in grinding processes requires precise constitutive equations describing dynamic material behaviour under extreme strain rates and large deformations. This study presents a novel methodology for optimising the Johnson–Cook (J–C) constitutive model parameters for micro-grinding applications, addressing the limitations of conventional mechanical testing at strain rates exceeding 10^5^ s^−1^. The research employed single abrasive grain micro-cutting experiments using a diamond Vickers indenter on aluminium alloy 7075-T6 specimens. High-resolution topographic measurements (130 nm lateral resolution) were used to analyse the scratch geometry and lateral material displacement patterns. Ten modified J–C model variants (A1–A10) were systematically evaluated through finite element simulations, focusing on parameters governing plastic strengthening (*B*, *n*) and strain rate sensitivity (*C*). Quantitative non-conformity criteria assessed agreement between experimental and simulated results for cross-sectional areas and geometric shapes of material pile-ups and grooves. These criteria enable an objective evaluation by comparing the pile-up height (*h*), width (*l*), and horizontal distance to the peak (*d*). The results demonstrate that conventional J–C parameters from Hopkinson bar testing exhibit significant discrepancies in grinding conditions, with unrealistic stress values (17,000 MPa). The optimised model A3 (*A* = 473 MPa, *B* = 80 MPa, *n* = 0.5, *C* = 0.001) achieved superior convergence, reducing the non-conformity criteria to Σ*k_A_* = 0.46 and Σ*k_K_* = 1.16, compared to 0.88 and 1.67 for the baseline model. Strain mapping revealed deformation values from *ε* = 0.8 to *ε* = 11 in lateral pile-up regions, confirming the necessity of constitutive models describing material behaviour across wide strain ranges. The methodology successfully identified optimal parameter combinations, with convergence errors of 1–14% and 7–60% on the left and right scratch sides, respectively. The approach provides a cost-effective alternative to expensive dynamic testing methods, with applicability extending to other ductile materials in precision manufacturing.

## 1. Introduction

Machine parts, especially those operating in tribological kinematic pairs, should have a well-defined surface texture related to their operating state. It is also important that the final surface-finishing step be performed with low machining energy [1], ensuring the minimisation of thermal- and mechanical-affected zones on the surface layer [2].

Experimental studies of material removal processes during machining do not provide a complete picture of all processes occurring around cutting edges. Many researchers investigate and apply the finite element modelling of machining processes to accurately explain the mechanisms of material removal phenomena [2,3,4]. The constitutive model parameters describe the dynamic mechanical behaviour of materials under cutting conditions. Selecting the correct parameter set is complex but critical, as it directly affects the accuracy of the simulation results.

The Johnson–Cook model is widely applied in the modelling and simulation of turning and milling. Simulation using these models has enabled the analysis of physical phenomena that influence the ways in which chips serrate [5,6,7]. Simulation studies have also enabled the prediction of the chip morphology across a broad spectrum of cutting speeds for aluminium alloy 7075-T651 [5] and enabled the determination of the effects of changes in the cutting edge rake angle on the chip form and size [6].

In the field of machining, particularly in the process of grinding, establishing the initial conditions for material removal remains a substantial challenge [8,9]. Numerous experimental and numerical investigations employ Johnson–Cook models in conjunction with destructive models, such as Cockcroft and Latham, incorporating modifications for strain softening scenarios. These models encompass power-law strain hardening, strain rate sensitivity, and polynomial thermal softening, as well as temperature-dependent damage models [10]. Notably, the highest level of simulation study agreement was achieved with a modified Johnson–Cook model that integrated strain hardening, thermal softening, and their interactions [11]. These simulation results enabled a comprehensive analysis of chip formation mechanisms, including the prediction of forces and temperatures during the cutting process [4].

In grinding processes, the analysis of the material removal process within the grinding zone is achieved through experimental research [12,13] and micro-grinding simulations employing a single abrasive grit. In contrast to turning or milling, the modelling of the material removal process by a single abrasive grit is more intricate. This is primarily attributed to the abrasive grit’s geometry, causing variations in chip formation initiation.

The cutting edges employed in machining have well-defined geometries, particularly those with positive rake angles. Conversely, abrasive grains are more effective in characterising intricate geometries, including those with negative rake angles. This distinction—among other factors, such as cutting forces, temperature, and energy consumption—is crucial in the machining process [14,15].

The geometric parameters of abrasive grits significantly influence the formation of flashes and chips during grinding. These parameters encompass the opening angle, rake angle, tip angle, and the orientation of the edges relative to the grinding direction [16]. These parameters exert a profound impact on each initiation step of the abrasive grit interacting with the material, including rubbing, ploughing, and chip formation. They also affect the effectiveness of the material removal ratio [17,18,19,20]. Similar conclusions are presented in papers [3,21], where the authors describe research studies on the cutting edge orientation and its impact on the material removal effectiveness.

**Figure 1:**
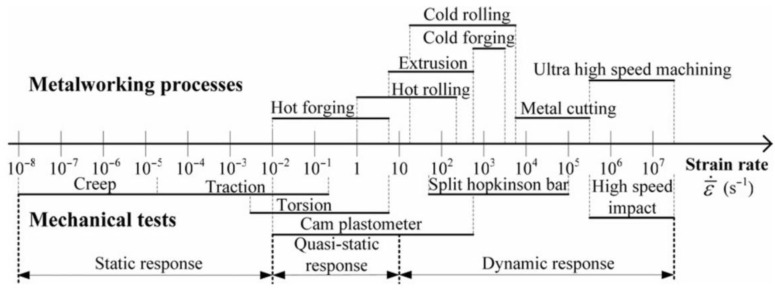
Strain rate ranges achieved in various metalworking processes and the corresponding response to material behaviour [23].

A notable distinction in the analysis of material removal across various machining techniques lies in the deformation speed values [22], within the contact region between the tool and the machined workpiece (Figure 1). Consequently, for machining processes, it is highly justified to investigate the dynamic material behaviour, as exemplified by the Split Hopkinson Pressure Bar (SHPB) method, to establish a constitutive model of the material.

The analysis of the state of the art (SOTA) revealed a limited number of research methods capable of obtaining data on the dynamic properties of metal alloys for deformation speeds $\dot{\varepsilon }$ exceeding 10^5^, particularly for grinding processes [24]. Understanding the state of deformation in the vicinity of abrasive grains is only feasible through the judicious selection of a constitutive model. This approach is contingent upon a high level of agreement between experimental and simulation results. The primary objective of this research was to develop a methodology for adjusting the Johnson–Cook model parameters, based on the assessment of parameters describing cross-sections of scratches resulting from the interaction of a single abrasive grain with the material. Additionally, the research aimed to determine the influences of the hardening effect and strain rate of the Johnson–Cook model on the lateral material flows during micro-machining with a single grain from a diamond Vickers indenter.

The research findings provide a basis for methodology development, enabling the selection of constitutive model parameters. The model describes the dynamic behaviour of metal alloys under typical grinding conditions characterised by high rates of material deformation.

## 2. Materials and Methods

### 2.1. Experimental Setup

In this study, aluminium alloy 7075 was utilised. The micro-grinding process was conducted using a Vickers indenter HV-3 (© HAHN+KOLB Werkzeuge GmbH, Ludwigsburg, Germany) with a tip angle of 136 degrees. The selection of a cutting edge with a defined geometry ensured the stability and repeatability of the process’ execution. The Vickers indenter provides a mathematically defined negative rake angle. This enables the precise calibration of the constitutive model under controlled conditions, which can then be applied to the random geometries of abrasive grains in grinding simulations. Prior to testing, the workpiece surface was ground to maintain a consistent depth of cut.

The micro-cutting process was conducted on a scratch test stand at a speed of 10 m/s. The process was repeated five times, and no significant changes in lateral material flows were observed.

**Figure 2:**
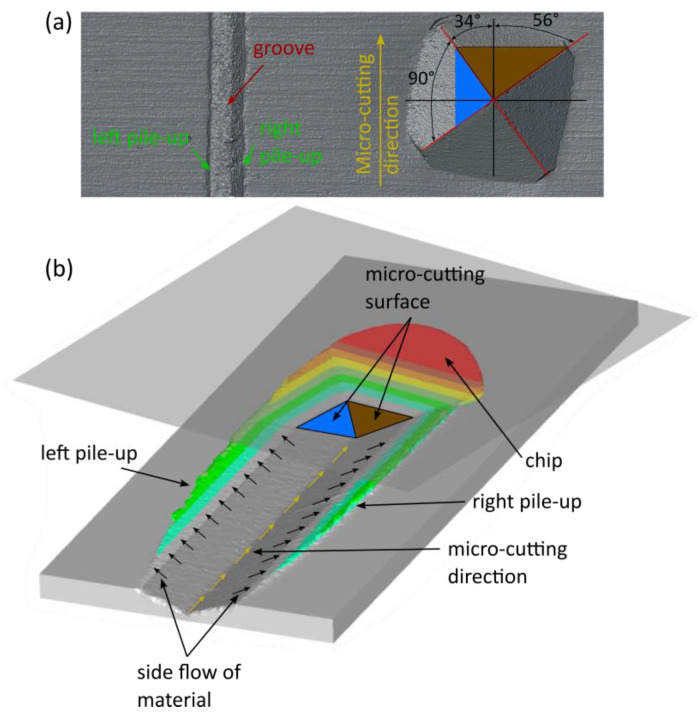
Scratch representation view (top view), where (**a**) is an in-cut created by the cutting grain (Vickers indenter) and (**b**) is its description (lower view).

**Figure 3:**
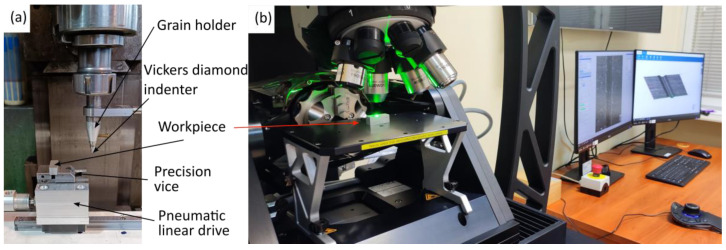
A comprehensive overview of the test stand designed for scratch tests, where (**a**) represents the scratch test stand and (**b**) the measurement system for surface and scratch analysis (Alicona G6).

The asymmetric orientation of the diamond grain relative to the cutting direction results in three effective cutting edges (Figure 2). Two edges, with cutting angles of 56 and 34 degrees, marked in brown, are responsible for chip formation. The edge cutting at 124 degrees, marked in blue, is responsible for material flow, resulting in side pile-ups (Figure 3). Data regarding the cutting grain geometry and its orientation towards the cutting direction were utilised in simulation studies.

**Figure 4:**
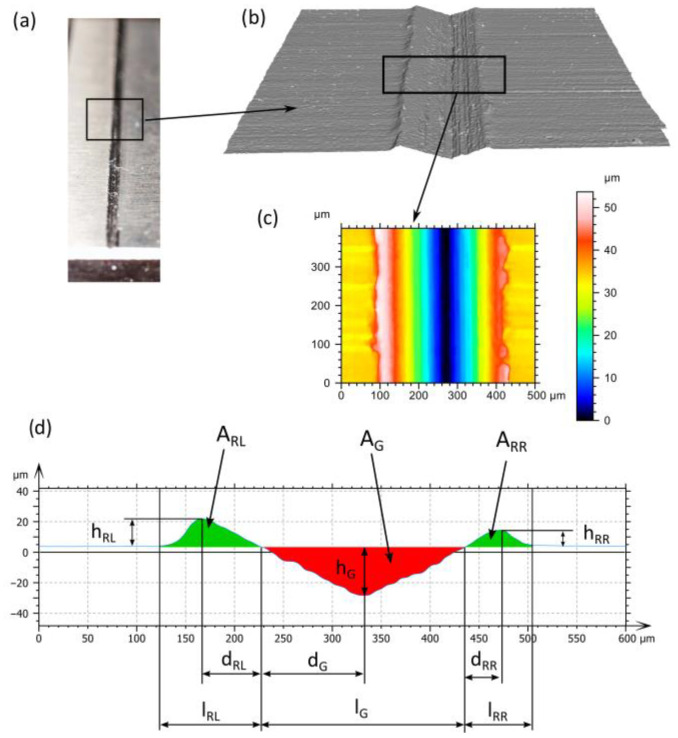
Experimental and simulation analysis of the scratch: (**a**) image view, (**b**) 3D photorealistic simulation, (**c**) heatmap top view, (**d**) cross-section profile and its associated parameters.

Topographic measurements of micro-scratches were conducted on the Bruker Alicona Focus Variation Microscope, Infinite Focus G6 (Alicona, Graz, Austria) (Figure 3b), employing the 800 WD37 objective. Each surface measurement was conducted with a lateral resolution of 130 nm, spanning dimensions of 1.6 mm by 1.6 mm. The measurement data were subsequently analysed using the SensoMap^®^ software v.11.01 (Figure 4), which is an OEM software tool provided by DigitalSurf^TM^ (Besançon, France). The average scratch profile length of 400 micrometres was analysed. The parameters that will be analyzed are presented in Table 1.

### 2.2. Constitutive Models

Simulation studies of material removal were conducted using the Johnson–Cook material model. The constitutive equations of this model have found application in the modelling of machining and abrasive processes, such as grinding. The model is also used in the modelling of materials subjected to deformation over a wide range of strain velocities and temperatures. The general form of the Johnson–Cook model, discussed and presented broadly in [3,17,18,25], is presented in Equation (1).

(1) $$\sigma =\left(A+B({\varepsilon_{p})}^{n}\right)\left(1+C\times ln\frac{{\dot{\varepsilon }}_{p}}{{\dot{\varepsilon }}_{0}}\right)\left(1-{\left(\frac{T-{\;T}_{amb}}{T_{melt}-T_{amb}}\right)}^{m}\right)$$

$$\sigma =\begin{array}{ccc} \underbrace{\left(A+B({\varepsilon_{p})}^{n}\right)} & \underbrace{\left(1+C\times ln\frac{{\dot{\varepsilon }}_{p}}{{\dot{\varepsilon }}_{0}}\right)} & \underbrace{\left(1-{\left(\frac{T-T_{amb}}{T_{melt}-T_{amb}}\right)}^{m}\right)}\\ strain\;hardening\;effects & strain\;rate\;effects & thermal\;softening\;effects \end{array}$$

where *A*—initial, static yield strength; *B*—parameter of plastic strength; *ε_p_*—effective plastic strain; *n*—exponent of plastic deformation strength; *C*—material parameter specifying the strain rate sensitivity of plastic speed deformation; $\dot{\varepsilon_{p}},$ $\dot{\varepsilon_{0}}$—effective plastic and reference strain rates; *T*, *T_amb_*, *T_melt_*—current, ambient, and melting temperatures; *m*—thermal softening exponent.

### 2.3. Simulation Assumptions and Selection Constants for the Johnson–Cook Model

Simulation studies were conducted using the Deform v14.0.2 software. Translational and rotational degrees of freedom were constrained for the nodes at the base of the workpiece. Boundary conditions were applied for the grain and aggregate velocities, which were set at 10 m/s. The following simplifications were introduced in the simulation: the physical properties of the grains were neglected, and they were modelled as rigid bodies, while the workpiece was modelled as homogeneous throughout its entire volume.

The modelling of material separation and displacement during grinding presents significant challenges due to the intricate interplay among numerous physical phenomena within the cutting zone and the complex contact between the cutting edges and the material. Notably, in grinding operations, the strain rates are substantially higher in magnitude compared to those observed in Hopkinson tests (Figure 1). These strain rate values serve as the primary data source for the development and refinement of constitutive models. The deformation values during grinding are directly proportional to the grinding speed (v) and inversely proportional to the cutting depth ($h_{s}$), as shown in Equation (2).

(2) $$\dot{\varepsilon }=\frac{v}{h_{s}}$$

In conventional grinding processes, the grinding speed typically ranges from a dozen to several dozens of meters per second. The cutting depth corresponds to the maximum chip thickness, which is the maximum undeformed chip thickness. Depending on the grinding parameters, the cutting depth can vary from a dozen to several dozens of micrometres. Consequently, the strain values generated during grinding can range from 10^5^ to 10^7^ s^−1^.

**Figure 5:**
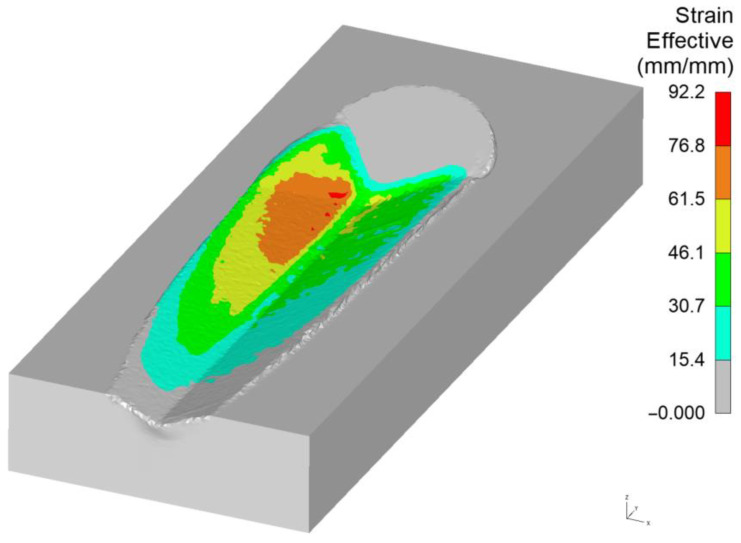
Deformation map in the material removal process employing a negative rake angle blade.

The strain values, which increase in proportion to the penetration depth and the length of the grain’s contact with the material, substantially exceed the typical strains observed in mechanical testing. The strain values reported in [26] and the authors’ studies (as depicted in Figure 5) demonstrate that constitutive models for grinding should be subjected to analysis for significantly higher strains, reaching up to 80 mm/mm.

**Figure 6:**
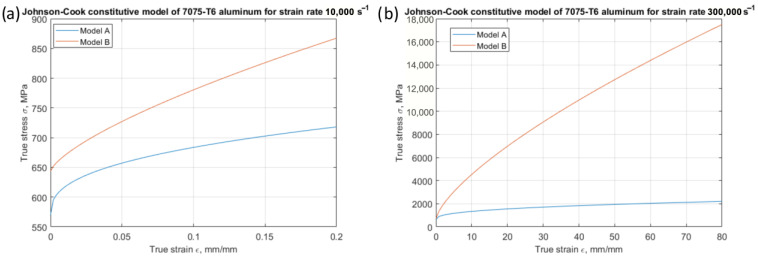
Stress–strain curves for models A and B in strain range for (**a**) turning and milling and (**b**) grinding.

The foundation for work with the constitutive model for the 7075-T6 aluminium alloy, which was utilised in the simulation, were models developed based on Hopkinson’s studies [27] (Model B, Figure 6) and based on studies conducted in a tensile test machine [28] (Model A, Figure 6). Both models’ parameters are presented in Table 2.

The stress–strain curves for both models, for strain rates of 10^4^ s^−1^ and a strain range of 0 to 0.2, are presented in Figure 6a. These strain rates and values are commonly employed during the development of constitutive models for turning and milling processes. A notable difference of 150 MPa is readily apparent.

In contrast, the same models exhibit distinct responses when applied to typical grinding processes, where the strain rates are approximately 3 × 10^5^ s^−1^ and the strain ranges from 0 to 80 (Figure 6b). Notably, the stress values for model B reach 17,000 MPa, significantly deviating from the actual behaviour of the aluminium alloy 7075-T6.

Model A was selected for further analysis due to its acceptable stress values within the large strain range. A parameter tuning process was conducted to optimise the Johnson–Cook model parameters (*B*, *n*, and *C*), as per Table 3. This tuning process enables the attainment of a high level of agreement in scratch geometry, even at high strain rates. The thermal softening exponent m was neglected in the analysis. In the process of lateral material displacement in the form of pile-ups, strain hardening dominates over thermal softening. This is attributed to the very short contact time and the ratio of the workpiece volume to the cut volume. Consequently, the parameter m was set to a value of 1 in the simulation studies.

**Figure 7:**
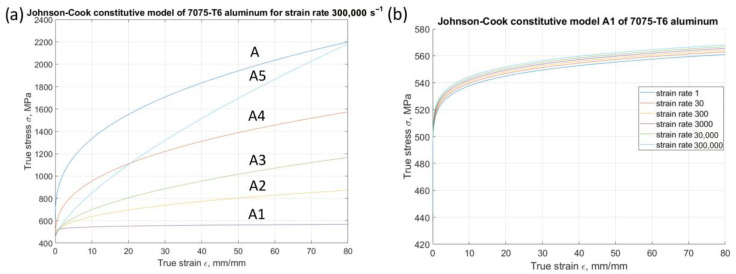
Stress–strain curves for modified constitutive models from A1 to A5. (**a**) For all models at strain rate 300,000 s^−1^; (**b**) model A1 for strain rates 1, 30, 300, 3000, 30,000, and 300,000 s^−1^.

In the analysis of models A1 to A5, a plastic deformation enhancement effect is observed on the material’s behaviour during single-grain micro-cutting (Figure 7a). In the A1 model, the influence of plastic deformation effects remains constant within the large deformation range. Conversely, in the A5 model, the influence exhibits a nearly linear change with increasing deformation. Models A2 to A4 represent intermediate states. Notably, the effect of varying deformation speeds on plastic deformation is not explicitly captured in the A1 to A5 models (Figure 7b). In these models, the parameter C was set to 0.001 to eliminate the influence of the strain rate on the shape of the curve.

**Figure 8:**
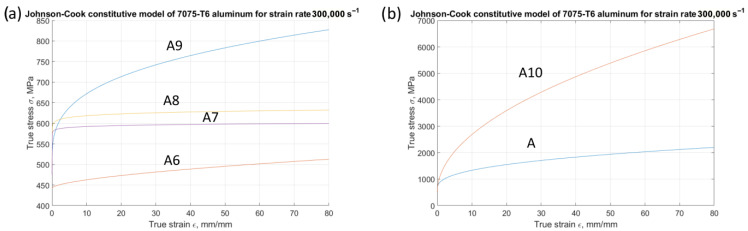
Stress–strain curves for modified constitutive models from A6 to A10. (**a**) For all models at strain rate 300,000 s^−1^; (**b**) for model A10.

Models A6 to A10 incorporate an analysis of strain rate variation (Figure 8). In these models, the constant value (*C*) exhibits a range, varying from 0.001 for model A6 (indicating a minimal strain rate effect, similar to models A1–A5) to 0.012 for model A10. These models consider the specific nature of plastic strengthening changes. Models A7 and A8 assume a constant influence of plastic strengthening for high deformation values (exponent values of *n* = 0.03 and *n* = 0.05). Conversely, models A6, A9, and A10 adopt an exponential increase in the factor associated with plastic strengthening (exponent values of *n* = 0.6, *n* = 0.3, and *n* = 0.5).

### 2.4. Methodology and Criteria for Assessing Non-Conformity in the Scratch Area, Depth, and Side Pile-Ups

Non-conformity criteria were formulated to quantify the extent of non-conformity between groove and material pile-up formation fields (Equations (3) and (4)). The primary objective of these criteria is to assess the appropriateness of the Johnson–Cook constitutive equation in fitting experimental results derived from single abrasive grain micro-machining processes. The parameters that delineate groove formation and material pile-up characteristics, as defined in the criteria, are presented in Section 2.1 (Table 1).

(3) $$k_{A_{i}}=\left|\left(1\;-\;\frac{A_{i_{s}}}{A_{i_{e}}}\right)\right|$$

where $A_{i_{s}}$—vector of parameters of the cross-sectional area of the groove or material pile-up from simulation; $A_{i_{e}}$—vector of parameters of the cross-sectional area of the groove or material pile-up from experiments; $i\;=\;\left[G\;RL\;RR\right]$; $i\;=\;\left[G\;RL\;RR\right]$.

(4) $$k_{ki}=\sum_{i=1}^{3}\left|1\;-\;\frac{j_{i_{s}}}{j_{i_{e}}}\right|$$

where $k_{ki_{s}}$—cross-sectional shape parameter of a groove or material pile-up from simulation; $k_{{ki}_{e}}$—cross-sectional shape parameter of a groove or material pile-up from experiments; $i\;=\;\left[G\;RL\;RR\right]$; $j\;=\;\left[l_{i}\;h_{i}\;d_{i}\;\right]$.

Criteria *k_A_* and *k_k_*, as defined in Equations (2) and (3), quantify the degree of non-conformity between the cross-sectional areas of material pile-up formation fields *A_RL_* and *A_RR_*, groove cross-section *A_G_*, and the cross-sectional area shape of the groove and material pile-up (length *l*, width *h*, depth *d*). A value further from 0 indicates a greater discrepancy in the parameters’ non-conformity, which characterises the groove area and shape as the result of the micro-cutting process. The cross-sectional area non-conformity criterion can be independently determined for the right pile-up $k_{A_{RR}}$, left pile-up $k_{A_{RL}}$, and groove $k_{A_{G}}$. Similarly, the criterion for groove cross-section shape non-conformity can be independently determined for the right pile-up $k_{K_{RR}}$, left pile-up $k_{K_{RL}}$, and groove $k_{K_{G}}$.

## 3. Results

**Figure 9:**
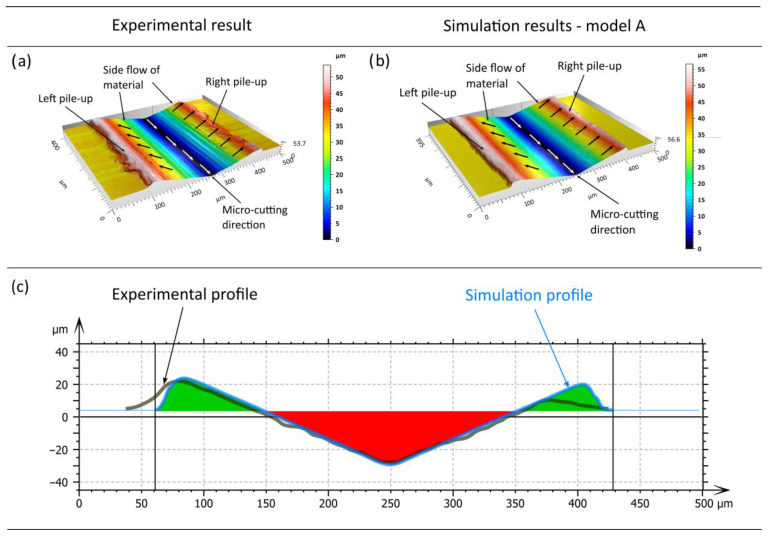
Results of scratch shape and surface measurements based on the average profile from (**a**) experimental studies, (**b**) simulation studies (model A [28]), and (**c**) comparison of analysed profiles.

The experimental data analysis commenced with the isolation of a 400 × 500 µm surface area from the micro-cutting scratch produced by a single diamond Vickers indenter of known geometry, as outlined in Section 2.4 (Figure 3). Based on the average profile (Figure 9), the cross-sectional areas and shapes of the groove and material pile-up were determined, and the results are presented in Table 4. The experimental data obtained and presented in Table 4 serve to validate the models described by the Johnson–Cook constitutive equation.

The grinding simulation, conducted using a diamond Vickers indenter to perform single-grain micro-cutting, was initially conducted for model A developed in [28]. The simulation results (model A) are presented in Figure 9 and Table 4, along with the corresponding experimental results.

An analysis of the areas and shapes of the lateral pile-ups reveals discrepancies between the results obtained from the experiments and simulation studies. The cross-sectional area of the left pile-up $A_{RL}$ differs only slightly. The simulated material pile-up exhibits a 3.6% increase in area compared to the measured one. Additionally, the width of the simulated left pile-up $l_{RL}$ is 15% smaller than the measured value. Conversely, on the right-hand side, there are noticeable differences. The simulated pile-up area on the right-hand side $A_{RR}$ is significantly larger, approximately 83.5% greater than the experimental value. This disparity is also evident in the shape of the pile-ups, which are defined by the parameters $l_{RR}$, $h_{RR}$, and $d_{RR}$.

Consequently, it can be postulated that the Johnson–Cook model parameters responsible for material strain hardening parameters are inaccurate, resulting in substantial resistance to lateral material flow, as evidenced by the pronounced pile-ups. The accuracy of a particular model is contingent upon parameters that are formulated based on mechanical testing outcomes, which provide insights into material behaviour under specific strain states and strain rates. Section 2.3 outlines the methodology for acquiring data for simulation studies. The input parameters were material data derived from strength tests, upon which the Johnson–Cook constitutive equation was developed [28].

Proposed modifications to the Johnson–Cook model facilitate the verification of the impact on the material removal mechanism and lateral material displacement during micro-cutting with a precisely defined geometry and orientation of abrasive grains. The Johnson–Cook model parameters, based on scratch tests, enable the more accurate development of a constitutive equation that accurately describes the material’s behaviour during grinding.

**Figure 10:**
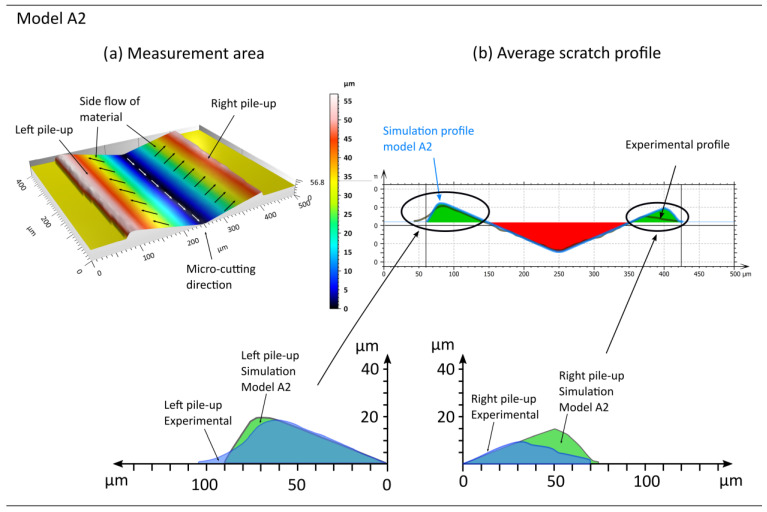
Example results obtained from the simulation conducted using model A2, where (**a**) represents the machined area and (**b**) the shape of the pile-ups, based on the analysis of the average cross-sectional profile.

**Figure 11:**
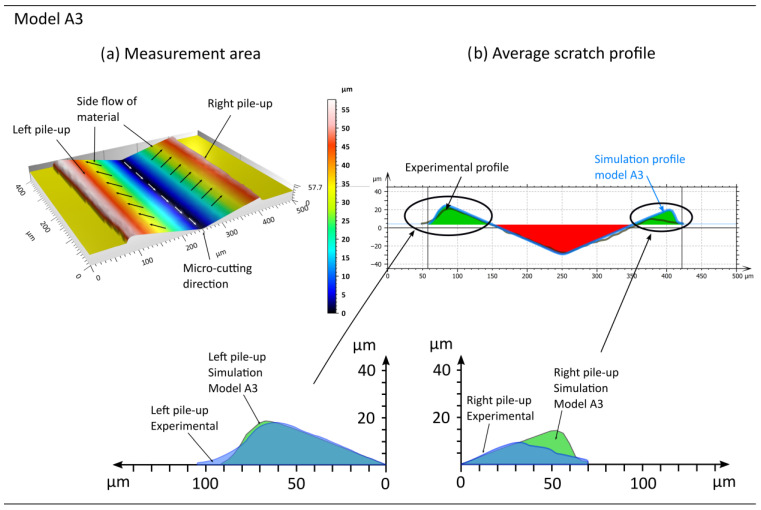
Example results obtained from the simulation conducted using model A3, where (**a**) represents the machined area and (**b**) the shape of the pile-ups, based on the analysis of the average cross-sectional profile.

The subsequent section of the article presents an analysis of the simulation results obtained from modified Johnson–Cook models A1 to A5. The primary objective of these studies was to investigate the impacts of parameters responsible for the plastic strain hardening of the material, excluding the influence of the strain rate (Figure 10 and Figure 11, Table 5).

Upon examining the lateral material flow on the left side, it is evident that the most satisfactory outcomes were achieved with models A3 and A2. The left pile-up area $A_{RL}$ obtained through simulation studies using model A2 is 0.5% larger.

Regarding the parameters that define the shape of the left pile-up, the results obtained using model A3 exhibit the most favourable performance in this comparison. The discrepancy in pile-up height $h_{RL}$ between the simulation and experiments is 6.6%. Additionally, the left pile-up width $l_{RL}$ and the width to the highest point of the left pile-up $d_{RL}$ are 12.5% and 5%, respectively.

The highest agreement between the pile-up area and shape was observed for model A3, situated on the right-hand side. The discrepancy between the experimental and simulated values for *A_RR_*, representing the pile-up area, is substantial, reaching a remarkable 40%. Notably, the width of the right pile-up, denoted as $l_{RR}$, observed experimentally corresponds to the simulated value. Conversely, the parameters describing the shape of the right pile-up, namely $d_{RR}$ and $h_{RL}$, exhibit significantly higher values in comparison to the simulation results, with respective increases of 35% and 24%.

The comparison in Table 6 reveals that both models (A6 and A9) achieve reasonable agreement for left-side pile-up parameters, with differences ranging from 11 to 14.4%. However, significant discrepancies exist for right-side pile-ups, particularly in the pile-up height (55–58% difference) and width to the highest point (32–34% difference). Model A9 shows slightly better performance for the right-side pile-up width (7.1% vs. 11.4% for A6), while both models perform similarly for left-side parameters. The asymmetric accuracy between the left and right sides suggests potential issues with material adhesion, built-up edge formation, or mesh resolution effects in the simulation.

**Figure 12:**
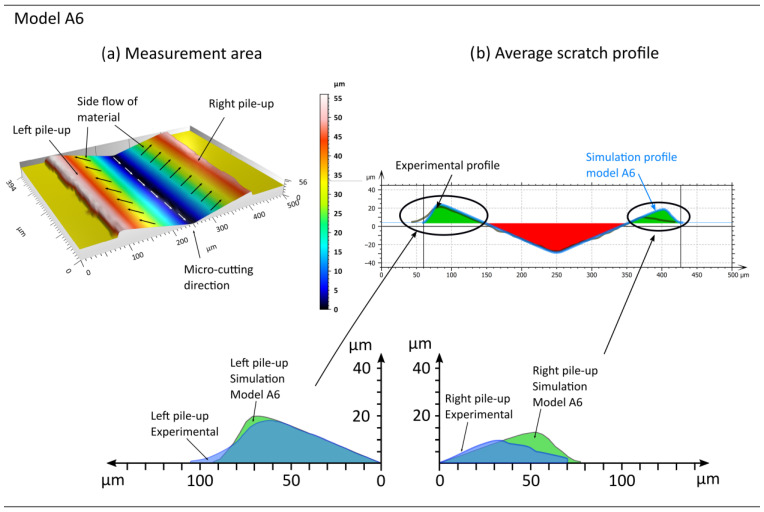
Example results obtained from the simulation conducted using model A6, where (**a**) represents the machined area and (**b**) the shape of the pile-ups, based on the analysis of the average cross-sectional profile.

**Figure 13:**
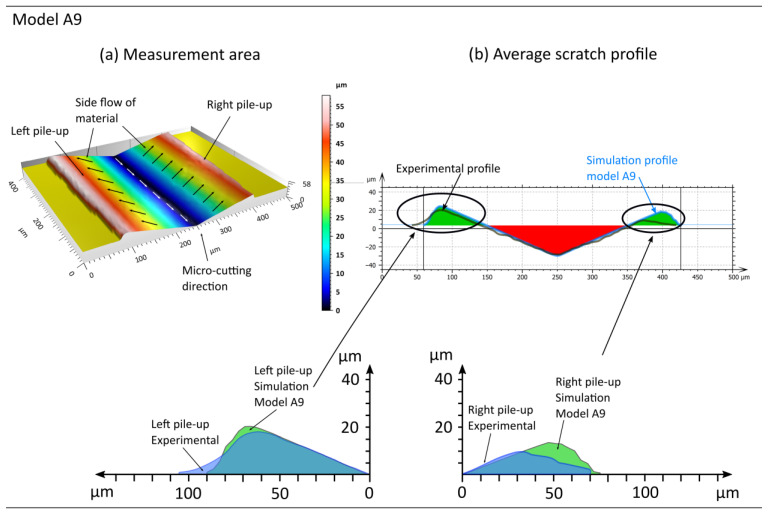
Example results obtained from the simulation conducted using model A9, where (**a**) represents the machined area and (**b**) the shape of the pile-ups, based on the analysis of the average cross-sectional profile.

In the subsequent phase, an analysis of the lateral material displacement results was conducted for models A6 to A10, which incorporated the influence of the strain rate on material hardening (Figure 12 and Figure 13, Table 7). Upon analysing the pile-ups obtained in the simulation studies, the most suitable fits can be observed for models A6 and A9. The discrepancies in left-side pile-up area $A_{RL}$ between the simulation studies and experiments are 2% for model A9 and 3% for model A6. Significantly poorer convergence of the pile-up area results was obtained on the right side $A_{RR}$, where the difference between the experiments and studies for models A6 and A9 is approximately 64% in both cases.

The presented methodology for measuring and analysing pile-ups and scratches demonstrates that, despite the good convergence of the pile-up cross-sectional area results, there exists significant divergence in their shapes. Consequently, information about cross-sectional areas is insufficient for the proper evaluation of lateral material displacements. Measuring the height *h*, width *l*, and width to the highest point *d* of pile-ups provides essential data for assessing the influence of changes in Johnson–Cook model parameters. These parameters are responsible for the plastic strengthening and plastic strain rate intensity of the material, as well as lateral flows of the machined material. The best convergence of the pile-up cross-sectional area results *A_RL_* and *A_RR_* and shape *l_RL_*, *l_RR_*, *d_RL_*, and *d_RR_* was obtained for models A3, A2, and A9, where the results’ convergence on the left side ranged from 1 to 14% and on the right side from 7 to 60%. It is noteworthy that models A3, A2, and A9 possess plastic strengthening curves of the material that exhibit a wide range of strain changes.

Consequently, it can be inferred that aluminium alloy 7075-T6 exhibits plastic deformation over a broad strain range during the micro-cutting process with high strain rates, as evidenced by the observed pile-ups.

Strength tests support the process of building constitutive models, but, due to the small range of strain rates shown in Figure 1, they may be insufficient to describe the behaviour of the material during the grinding process. These studies are primarily used to determine threshold values of parameters for the evaluation of a specific set of models. Consequently, it is justified to employ the non-conformity criterion for cross-sectional areas, the pile-up shape, and grooves. These criteria facilitate the verification of the accurate alignment of the Johnson–Cook model parameters in micro-cutting processes involving a single abrasive grain, thereby ensuring the applicability of the model to strain rates observed in grinding processes.

**Figure 14:**
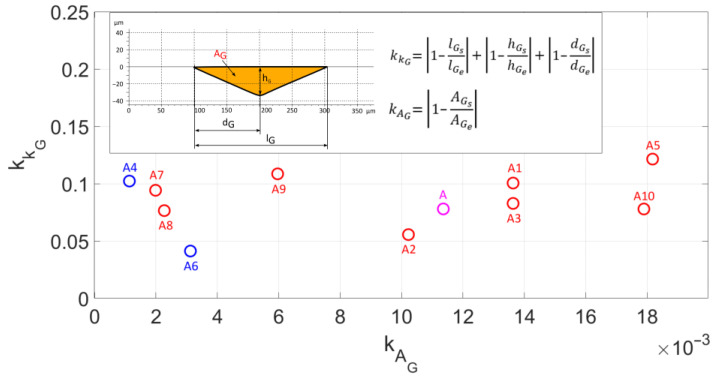
The criterion for non-compliance of the cross-sectional area $k_{A_{G}}$ and the shape $k_{K_{G}}$ of the scratch.

Figure 14 presents the non-conformity criteria for cross-sectional area $k_{A_{G}}$ as well as scratch shape $k_{K_{G}}$. The values of the non-conformity criterion for fitting the Johnson–Cook model parameters from simulation studies to experimental study results indicate the correct orientation of the grain geometry in the cutting direction. Small differences in the results indicate the elastic–plastic and plastic behaviour of the material in the micro-cutting process. The non-conformity criteria for the cross-sectional area and groove shape for model A [28] were $k_{A_{G}}=0.011$, $k_{K_{G}}=0.078$, respectively. Among all analysed modified models, models A4 and A6 exhibit the best fitting of the simulation study results to experimental studies. The best fitting based on the scratch surface area non-conformity criterion belongs to model A4 and equals $k_{A_{G}}=0.0011$, while the criterion describing the scratch shape is the best for model A6 and equals $k_{K_{G}}=0.041$.

Upon further examination of the discrepancies in the non-conformity outcomes for the cross-sectional area and scratch morphology, it is evident that the damage model proposed by Cockcroft and Latham was appropriately selected. Material separation manifests when the damage coefficient attains a critical threshold. In the simulation studies, this threshold was established at *D_f_* = 300.

**Figure 15:**
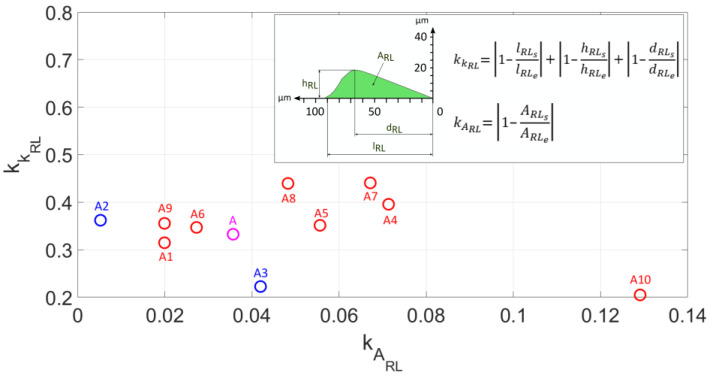
The criterion for non-compliance of the cross-sectional area $k_{A_{L}}$ and the shape $k_{K_{L}}$ of the left-side pile-up.

Figure 15 presents the criteria for non-conformity of the cross-sectional area $k_{A_{RL}}$, as well as the shape $k_{K_{RL}}$, of the left pile-up. As previously mentioned, experimental analysis of lateral material displacements in grinding processes is practically impossible, and information about the state of strain and stress in these zones is important in accurately describing the formation of the geometric structures of technological surfaces. The best fit of the Johnson–Cook model parameters was demonstrated by model A2, where the non-conformity criterion for the left pile-up cross-sectional area was $k_{A_{RL}}=\;0.0052$, and by model A3, for which the non-conformity criterion for the shape of the left pile-up was $k_{K_{RL}}=\;0.22$.

**Figure 16:**
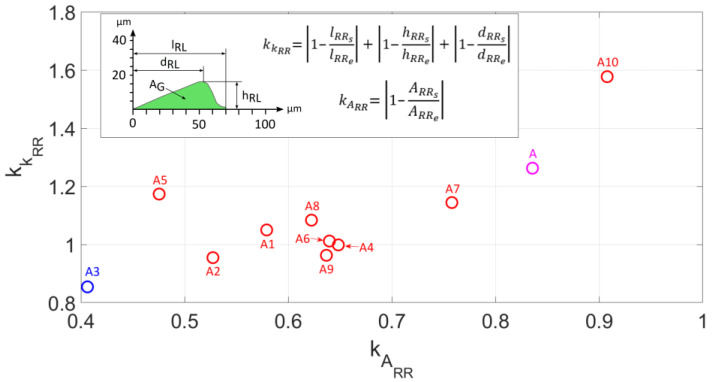
The criterion for non-compliance of the cross-sectional area $k_{A_{L}}$ and the shape $k_{K_{L}}$ of the right-side pile-up.

The non-conformity criteria for the cross-sectional area as well as the right pile-up shape presented in Figure 16 demonstrate that model A3 achieves the best fitting of the Johnson–Cook model parameters. The non-conformity criterion for the right pile-up cross-sectional area is $k_{A_{RR}}=0.41$ and that for the right pile-up shape is $k_{K_{RR}}=0.85$.

**Figure 17:**
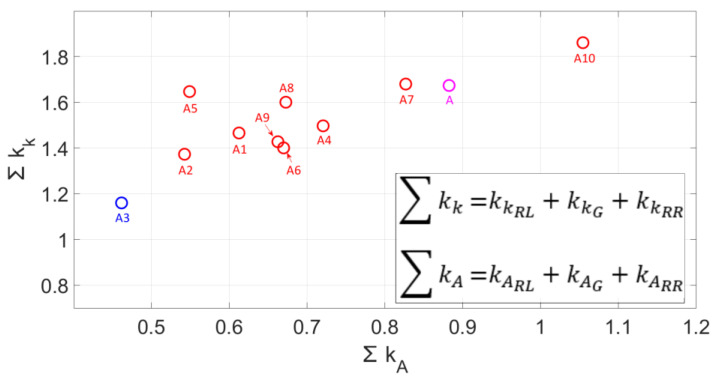
The sum of the criteria of non-conformity of the surface $k_{A}$ and the shape $k_{K}$ of the scratch and side pile-ups.

Figure 17 illustrates the summation of the criteria for the inconsistency in the cross-sectional area of the right pile-up $k_{A_{RR}}$ and left pile-up $k_{A_{RL}}$, as well as the scratch $k_{A_{G}}$. Similarly, it depicts the shapes of the right pile-up $k_{K_{RR}}$ and left pile-up $k_{K_{RL}}$ and the scratch $k_{K_{G}}$. A lower criterion value for the fit of the Johnson–Cook model parameters between the simulation and experiment results in the greater convergence of the surfaces and shapes of the pile-ups and scratches.

Model A [28] demonstrates significant inconsistency in the pile-up area and scratch $\sum k_{A}=\;0.88$ and in the pile-up shape and scratch $\sum k_{K}=\;1.67$. Among all analysed and modified models, model A3 exhibits the optimal fit of the simulation results to experimental tests. The sum of the criteria for non-compliance in the cross-sectional area of pile-ups and scratches for model A3 is $\sum k_{A}=\;0.46$, and the sum of the criteria describing the lateral pile-ups and scratches is $\sum k_{K}=\;1.16$.

The proposed methodology for fitting the Johnson–Cook model parameters, based on the criterion of assessing the non-conformity of the areas and shapes of lateral pile-ups and scratches, facilitates inexpensive modifications of constitutive equations with a high degree of convergence to a specified machining process. This approach ensures that experimental results are not only utilised for the validation of the computer model. Employing the criteria of non-conformity of the cross-sectional areas of pile-ups and scratches *A_i_*, and their respective shapes *K_i_*, it is possible to swiftly identify minute alterations in model parameters responsible for, for instance, the plastic strengthening of the material. An analysis of the results indicates that, in the context of micro-cutting processes involving single grains with negative rake angles and high strain rates, it is advantageous to assess them using criteria that explicitly delineate the geometries of the left and right material pile-ups, as well as the scratch. The more closely the result of a given non-conformity criterion approaches zero, the more accurate the fit of the constitutive equation parameters describing the machined material.

## 4. Discussion

Modelling grinding processes using a single abrasive grain and employing the finite element method necessitates an understanding of the strain states and strain rates occurring during material removal. This knowledge enables the construction of constitutive models with significantly enhanced accuracy, accurately describing the flow of the machined material around the cutting edges of abrasive grains. The proper development of constitutive equations, such as the Johnson–Cook equation, requires measurements and analyses that facilitate the establishment of model parameters describing the dynamic properties of metal alloys for a broad spectrum of strain rates. The quasi-static or dynamic mechanical testing of materials alone cannot provide comprehensive information regarding dynamic material behaviour, particularly in strain rate ranges that extend beyond the scope of these tests. It is also crucial to consider strain states, which, due to the negative rake angles of abrasive grains, can be significantly higher than those observed during mechanical testing.

**Figure 18:**
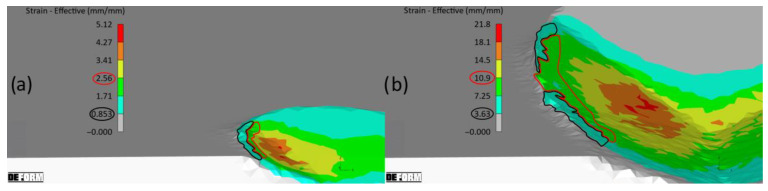
Strain maps of the left pile-up: (**a**) beginning of micro-cutting, (**b**) after travelling a micro-cutting distance of 0.3 mm.

Figure 18 shows the change in strain state in the left pile-up, with values ranging from *ε* = 0.8 to *ε* = 11. The strains in the zone of lateral material displacement increase with the increasing penetration of the grain into the material. Therefore, it is justified to use the scratch test, with strain rates typical for grinding, to tune the parameters of constitutive models describing dynamic material behaviour.

**Figure 19:**
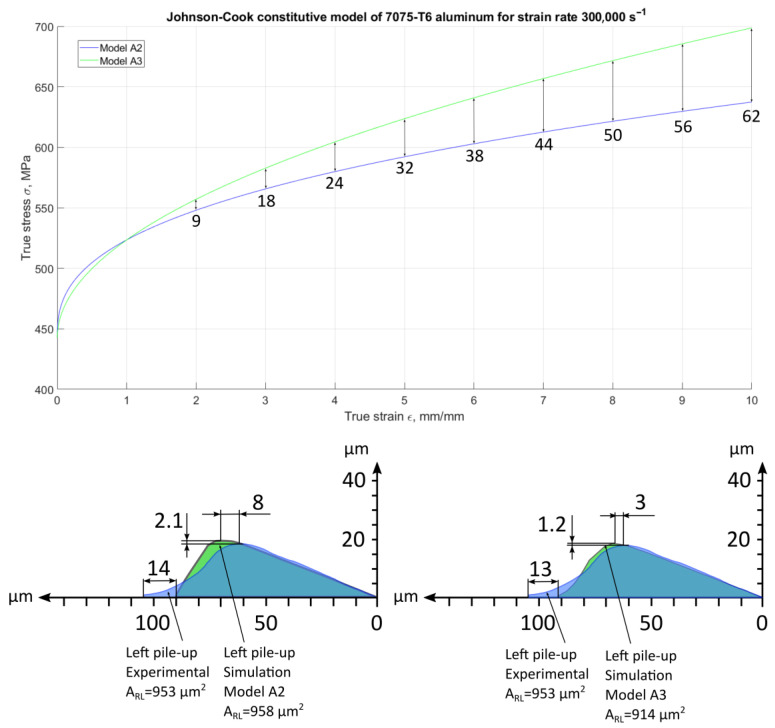
Models with the best fit for left-side pile-ups.

The optimal model for left-side pile-up convergence was identified as model A2, with a difference in lateral area ∆*A_RL_* of 5 µm^2^. Model A3, on the other hand, exhibited the best fit for the left pile-up shape, characterised by ∆*l_RL_* = 13 µm, ∆*d_RL_* = 3 µm, and ∆*h_RL_* = 1.2 µm (Figure 19).

As depicted in Figure 18, initial pile-up formation occurs within the strain range (*ε*) from 0 to 2. Within this strain range, models A2 and A3 do not exhibit substantial discrepancies. However, as the cutting progresses, material strengthening accompanies significant deformation, leading to strains exceeding *ε* = 10.

Model A3, in the strain range (*ε*) from 2 to 10, exhibits a greater degree of hardening compared to model A2. Specifically, within the stress range (*σ*) from 9 to 62, model A3 undergoes a significant transformation (Figure 19).

Consequently, it can be concluded that the accurate modelling of lateral material displacements and the fitting of the lateral pile-up shape are contingent upon the selection of a suitable constitutive model that accurately captures the material hardening state under the influence of high strain rates within a large strain range.

The asymmetric orientation of the abrasive grain in the micro-cutting direction resulted in material displacement along edges of varying lengths. The shorter cutting edge, due to the adhesion of the machined material, may form built-up edges, causing significant shape changes. Conversely, the longer cutting edge leads to increased cutting resistance, and the displaced machined material may remove built-up edges. Consequently, the influence of the cutting edge geometry, particularly the adhesion of the machined material to the tool edge (not included in the experimental model), significantly impacts the accuracy of pile-up shape modelling.

Aluminium alloy 7075-T6, a material prone to wheel loading and causing difficulties in cutting processes such as turning, milling, or threading, may have influenced the experimental studies. Built-up edges could have limited lateral material displacement, resulting in the separation of a larger portion of material in the form of chips.

The finite element mesh size employed in the simulation studies varied between 4 and 6 micrometres. For smaller pile-ups, this could have influenced the interpolation of their shapes, potentially resulting in shape inaccuracies in the right-side pile-up of the groove formed during the simulation studies.

## 5. Conclusions

The development of a constitutive equation necessitates the description of material behaviour during the micro-cutting process under conditions typical for grinding, as well as knowledge of the strain states and strain rates that occur during material removal. Mechanical testing methods, owing to their limitations regarding strain rates, pose a challenge in the development of a model described by a constitutive equation. The proposed methodology not only facilitates the deeper comprehension of processes occurring near cutting edges but also, based on experimental studies of single abrasive grain operation, facilitates the tuning of simulation model parameters with a high degree of accuracy and convergence. The authors anticipate that the described methodology will also work for other materials. Tuning the model parameters for brittle or ductile materials will require a different approach due to the different nature of the changes in their plastic properties under high strain rates.

The outcomes of the experimental and simulation studies pertaining to the optimisation of the Johnson–Cook constitutive equation parameters led to the following conclusions:

- The development of the Johnson–Cook constitutive model for dynamic material behaviour in the grinding process necessitates the verification of the accuracy of model parameter selection within the strain rate range of 10^5^ to 10^7^ and large strains induced by negative rake angles of abrasive grains, reaching *ε* = 70. Dynamic changes in the conditions within the contact zone between abrasive grains and the workpiece significantly affect the roughness parameters of ground surfaces. The methodology for tuning constitutive models makes it possible to build more accurate simulations, which will enable a better understanding of the surface formation process during grinding.
- An analysis of the simulation studies revealed that, while the pile-up cross-sectional area results exhibited satisfactory convergence, their actual shapes significantly deviated from reality. Comprehensive information regarding lateral material displacements during the micro-cutting process can be obtained by analysing the shapes of pile-ups, which were described in the studies as height *h*, width *l*, and width to the highest point *d*. Based on this information, the influence of tuning the Johnson–Cook constitutive model parameters on the convergence of computer simulation models with experimental studies can be estimated.
- The optimal convergence of pile-up cross-sectional area *A_RL_* and *A_RR_*, as well as the shape *l_RL_*, *l_RR_*, *d_RL_*, *d_RR_*, was achieved by modifying the Johnson–Cook model parameters *B* and *n*, which govern the material’s plastic strengthening curve, and parameter *C*, which accounts for the strain rate effects. The most favourable results were obtained for model A3, whose model parameters describing aluminium alloy 7075-T6 were as follows: *A* = 473 MPa, *B* = 80 MPa, *n* = 0.5, *C* = 0.001.
- The scratch test, conducted experimentally and computationally with velocities typical for grinding processes, employs a criterion to evaluate the non-conformity of the areas and shapes of lateral pile-ups and scratches. This test facilitates the tuning of the Johnson–Cook constitutive model parameters. An analysis of the criterion revealed that the optimal convergence of the simulation study results with experimental data was achieved for model A3. In this model, the results of the summation of the non-conformity criteria for both the pile-up and groove cross-sectional areas $\sum k_{A}=\;0.46$, as well as the summation of deviations in parameters defining the shape of lateral pile-ups and scratches $\sum k_{K}=\;1.16$, were the most effective. The proposed methodology reduced the mismatch criterion ($\sum k_{A}$) from 0.88 (baseline model A) to 0.46 (optimised model A3), representing a 47% improvement in volumetric accuracy.

## Figures and Tables

**Table 1:** Detailed description of the material’s side pile-up parameters and the scratch geometry.

$l_{RL}$	left pile-up width
$l_{RR}$	right pile-up width
$h_{RL}$	left pile-up height
$h_{RR}$	right pile-up height
$d_{RL}$	width to the highest point of the left pile-up
$d_{RR}$	width to the highest point of the right pile-up
$l_{G}$	groove width
$h_{G}$	groove height
$d_{G}$	width to the lowest point of the groove
$A_{RL}$	left pile-up area
$A_{RR}$	right pile-up area
$A_{G}$	groove area

**Table 2:** Parameters of Johnson–Cook constitutive model for 7075-T6 aluminium alloy [28,29].

Model	*A* [MPa]	*B* [MPa]	*n*	*C*
A [28]	473	210	0.3813	0.033
B [29]	527	575	0.71	0.024

**Table 3:** Parameters of Johnson–Cook constitutive model of 7075-T6 aluminium alloy model A [28] and its modifications from A1 to A10.

Model	*A* [MPa]	*B* [MPa]	*n*	*C*
A	473	210	0.3813	0.033
A1	473	80	0.1	0.001
A2	473	80	0.3813	0.001
A3	473	80	0.5	0.001
A4	473	210	0.3813	0.001
A5	473	80	0.7	0.001
A6	473	5	0.6	0.001
A7	473	100	0.03	0.007
A8	473	100	0.05	0.01
A9	473	80	0.3	0.01
A10	473	600	0.5	0.012

**Table 4:** Results of scratch shape and surface measurements derived from the average profile (experiment and model A [28]).

No.	*A_RL_* [µm^2^]	*l_RL_* [µm]	*d_RL_* [µm]	*h_RL_* [µm]	*A_RR_* [µm^2^]	*l_RR_* [µm]	*d_RR_* [µm]	*h_RR_* [µm]	*A_G_* [µm^2^]	*l_G_* [µm]	*h_G_* [µm]	*d_G_* [µm]
EXP	953	104	62	18.0	347	70	38	9.23	3521	205	31.7	103
A	919	88	68	19.8	637	73	54	16.6	3561	202	33.4	102

**Table 5:** The measurement outcomes of the pile-up area and its shape, derived from the average cross-sectional profile, from simulation models A1–A5 and experiment EXP.

No.	*A_RL_* [µm^2^]	*l_RL_* [µm]	*d_RL_* [µm]	*h_RL_* [µm]	*A_RR_* [µm^2^]	*l_RR_* [µm]	*d_RR_* [µm]	*h_RR_* [µm]	*A_G_* [µm^2^]	*l_G_* [µm]	*h_G_* [µm]	*d_G_* [µm]
A1	934	91	68	20	548	72	51	15.5	3569	202	33.5	100
A2	958	90	70	20.1	530	74	50	14.6	3557	204	33	102
A3	913	91	65	19.2	488	70	50	14.2	3569	203	33.4	101
A4	1021	95	71	21.3	572	71	50	15.4	3525	201	33.4	100
A5	900	90	69	20.2	512	71	55	15.8	3585	201	33.7	99
EXP	953	104	62	18.0	347	70	38	9.23	3521	205	31.7	103

**Table 6:** Percentage differences between experimental and simulated parameters.

Parameter	Side	Model A6	Model A9
Pile-up height (*h*)	Left	12.5%	14.4%
	Right	58.0%	55.0%
Pile-up width (*l*)	Left	12.9%	11.2%
	Right	11.4%	7.1%
Width to highest point (*d*)	Left	~11%	~11%
	Right	32%	34%

Note: All values represent the percentage difference between experimental and simulated results.

**Table 7:** The measurement outcomes of the pile-up area and its shape, derived from the average cross-sectional profile, from simulation models A6–A10 and experiment EXP.

No.	*A_RL_* [µm^2^]	*l_RL_* [µm]	*d_RL_* [µm]	*h_RL_* [µm]	*A_RR_* [µm^2^]	*l_RR_* [µm]	*d_RR_* [µm]	*h_RR_* [µm]	*A_G_* [µm^2^]	*l_G_* [µm]	*h_G_* [µm]	*d_G_* [µm]
A6	927	91	70	20	569	78	50	14.6	3532	205	32.7	102
A7	1017	92	72	21.3	610	76	52	15.6	3514	202	33.3	100
A8	999	91	71	21.4	563	71	52	15.7	3513	203	33.2	101
A9	934	89	69	20.1	568	75	51	14.3	3542	201	33.6	100
A10	830	90	65	18.7	662	79	59	17.5	3584	204	33.4	101
EXP	953	104	62	18.0	347	70	38	9.23	3521	205	31.7	103

## Data Availability

The data presented in this study are openly available in RepOD at https://doi.org/10.18150/QZK8FX, reference number QZK8FX.
